# A nine-country study of the protein content and amino acid composition of mature human milk

**DOI:** 10.3402/fnr.v60.31042

**Published:** 2016-08-26

**Authors:** Ping Feng, Ming Gao, Anita Burgher, Tian Hui Zhou, Kathryn Pramuk

**Affiliations:** 1 Wyeth Nutrition, Shanghai, China; 2 Wyeth Nutrition, King of Prussia, PA, USA; 3 Bio TX Clinical Research, Pfizer, Inc., Collegeville, PA, USA

**Keywords:** human milk, amino acid, protein, nitrogen, lactation stage

## Abstract

**Background:**

Numerous studies have evaluated protein and amino acid levels in human milk. However, research in this area has been limited by small sample sizes and study populations with little ethnic or racial diversity.

**Objective:**

Evaluate the protein and amino acid composition of mature (≥30 days) human milk samples collected from a large, multinational study using highly standardized methods for sample collection, storage, and analysis.

**Design:**

Using a single, centralized laboratory, human milk samples from 220 women (30–188 days postpartum) from nine countries were analyzed for amino acid composition using Waters AccQ-Tag high-performance liquid chromatography and total nitrogen content using the LECO FP-528 nitrogen analyzer. Total protein was calculated as total nitrogen×6.25. True protein, which includes protein, free amino acids, and peptides, was calculated from the total amino acids.

**Results:**

Mean total protein from individual countries (standard deviation [SD]) ranged from 1,133 (125.5) to 1,366 (341.4) mg/dL; the mean across all countries (SD) was 1,192 (200.9) mg/dL. Total protein, true protein, and amino acid composition were not significantly different across countries except Chile, which had higher total and true protein. Amino acid profiles (percent of total amino acids) did not differ across countries. Total and true protein concentrations and 16 of 18 amino acid concentrations declined with the stage of lactation.

**Conclusions:**

Total protein, true protein, and individual amino acid concentrations in human milk steadily decline from 30 to 151 days of lactation, and are significantly higher in the second month of lactation compared with the following 4 months. There is a high level of consistency in the protein content and amino acid composition of human milk across geographic locations. The size and diversity of the study population and highly standardized procedures for the collection, storage, and analysis of human milk support the validity and broad application of these findings.

Human milk is considered the best source of nutrition for term infants. The World Health Organization recommends exclusive breast feeding during the first 6 months of life (1). Within the nutrient-rich matrix of human milk, the quantity and quality of protein are vitally important to provide the infant with a source of peptides, amino acids, and nitrogen for visceral protein synthesis, tissue accretion, and growth. Additionally, human milk provides the amino acids required to synthesize hormones, enzymes, antibodies, and other compounds such as glutathione, nucleotides, and some neurotransmitters (2).

Numerous studies have evaluated protein and amino acid levels in human milk. The earliest studies yielded widely divergent findings that were attributed to variability among donors with respect to age, parity, and duration of lactation, as well as differences in the collection and storage of human milk samples, and methods of analysis (3). The introduction of the automated amino acid analyzer (4) represented a clear improvement in methodology that resulted in more consistent data on the protein and amino acid composition of human milk (5–21). Despite such advances in analytical methods, research on the protein and amino acid content of human milk has been limited by small sample sizes and homogeneous study populations. Moreover, studies differ with respect to sample collection, storage, and methods of analysis, all of which can introduce variability to the measurement of protein and amino acid levels in human milk.

To our knowledge, this study is the largest, multinational study of protein levels and amino acid composition in human milk. A review of the published literature of total protein and amino acid composition of human milk from various regions and varying collection techniques is summarized in Table 1. In our study, milk samples from 220 women from nine countries across five continents were analyzed for amino acid composition, total nitrogen, and true protein concentration, using a unified protocol and standardized methodology. Therefore, the potential variability inherent from any differences in the sample collection, handling, storage, shipping procedures, and sample analyses was essentially eliminated, thereby increasing our confidence that the variations in our data reflect true biological variations among the samples. Enrolling mothers over a broad range of days, post-partum, permitted the assessment of amino acid and protein levels across several stages of lactation. It has been shown previously that the protein level and amino acid content in human milk decrease over the course of lactation (20), whereas maternal race/ethnicity, age, and maternal dietary protein intake appear to have little effect on the total protein in human milk (22).

**Table 1:** Referenced studies of human milk amino acid and/or total protein composition

Authors	Location	Number of mothers	Lactation time	Reference	Total protein (g/L)^a^
Lonnerdal et al.	Sweden	6	2–3 months	(5)	10.7+1.34
Raiha et al.	Italy	10	Mature	(6)	12.0
Picone et al.	USA	12 pooled	Mature	(7)	(AA)
Atkinson et al.	Canada	8 pooled	Mature	(8)	(AA)
Renner	Germany	N/S	N/S	(9)	8.5 (no SD)
USDA	N/S	N/S	N/S	(10)	10.3
Svanberg et al.	Sweden	8	2–5 months	(11)	10.1+0.88
	Ethiopia	8	2–5 months	(11)	11.4+2.84
Jarvenpaa et al.	Sweden	Pooled	N/S	(12)	9.6
Harzer and Bindels	Germany	N/S	36 days	(13)	11.0
Donovan and Lonnerdal	USA	5	Mid-lactation	(14)	10.4+0.64
Hanning et al.	N/S	N/S	28–30 days	(15)	10.0
Motil et al.	USA	24	1–12 months	(17)	10.6+1.34
Darragh	New Zealand	20	10–14 weeks	(18)	11.5+0.19 (SE)
Zhao et al.	China	91	1–6 months	(19)	11.8+0.14
Wu et al.	Taiwan, China	105	46–297 days	(20)	12.6^b^ (no SD)
Feng et al.	Nine countries	220	30–188 days	Current study	11.9+0.20^c^

N/S, not specified; AA, amino acid composition only.

a Total protein=total nitrogen×6.38 for most studies (some values derived from biochemical assay).

b Total protein only includes samples with lactation days between 46 and 297 days.

c Mean total protein concentration in human milk calculated using 6.25 as the factor=11.9 (range 8.5–22.9 g/L); mean total protein concentration in human milk calculated using 6.38 as the factor=12.2 (range 8.9–23.4 g/L).

By using a single commercial entity for shipping, a single commercial laboratory for sample storage, a single research laboratory for sample analysis, and standardized milk collection procedures, our methodology was highly consistent across sites and assures a reliable data set.

## Materials and methods

### Study design and subjects

The human milk samples were collected as part of a cross-sectional survey of major carotenoids (23) and fatty acids (24) in human milk from healthy, well-nourished lactating women in nine countries: Australia, Canada, Chile, China, Japan, Mexico, the Philippines, the United Kingdom, and the United States. All participants were aged 18–40 years; were mothers of a healthy, full-term singleton infant; and were between 1 and 12 months postpartum at the time of milk collection. Participants signed written, informed consent in their native language prior to enrollment in the study, and the same two individuals conducted on-site training for all study personnel. The study was conducted in accordance with the principles of the Declaration of Helsinki and was approved by the Human Subjects Committee of the University of Arizona and the Human Ethics Committees associated with each participating institution.

A minimum of 50 human milk samples were collected from each of the nine countries represented in the study, for a total of 509 samples. Of these, 445 samples were from women who were 30–188 days (1–6 months) postpartum at the time of milk collection. Within this subset of 445 human milk samples, 220 were randomly selected for an analysis of amino acid and nitrogen composition. The random selection of samples was stratified by country to ensure the inclusion of at least 20 samples from each country.

### Collection and handling of human milk samples

Our collection and handling of the human milk samples has been described in detail (23). Briefly, a complete breast expression containing a minimum of 50 mL of human milk was collected between 1:00 p.m. and 5:00 p.m. on the day of the sampling. In all countries except Japan, samples were collected using an electric pump. In Japan, where the use of an electric pump is considered unacceptable to women, samples were collected manually using a hand-held breast pump under the supervision of clinic staff. After collection, milk samples were immediately placed on dry ice or in a freezer at −20°C, then shipped within 10–14 days on dry ice to a central laboratory where the samples were stored at −70°C. Prior to analysis, frozen samples from a single country were thawed overnight in the refrigerator. Under subdued lighting conditions, samples were warmed to 37°C in a water bath and gently stirred, and approximately 10 mL of each sample was transferred to a tube and stored at −70°C for a group analysis. One day before amino acid and nitrogen analyses, samples were thawed overnight in the refrigerator. Thawed samples were warmed to 37°C in a water bath and gently stirred, before subsequent analysis. The analysis of samples was grouped daily by randomly choosing two samples from each country to eliminate day-to-day bias.

### General amino acid analysis

To determine the concentration of 16 amino acids in the human milk samples, 10 mL of 6 M HCl (containing 0.1% phenol) was added to a hydrolysis tube that contained 1 mL of human milk. Following vacuum and nitrogen flush, repeated three times, the tube was sealed under a nitrogen blanket and the sample hydrolyzed by placing in an oven at 110°C for 24 h. The hydrolysate was then quantitatively transferred to a volumetric flask and made up to a volume of 50 mL using distilled water. A total of 15 µL of filtered hydrolysate solution was quantitatively pipetted into a derivatization tube, dried under vacuum, and then combined with alpha-amino butyric acid as an internal standard and analyzed using AccQ-Tag (Waters Corporation, Milford, MA) derivatization and high-performance liquid chromatography (HPLC) (25, 26).

### Cysteine analysis

Distilled water was added to 1 mL of the milk sample in a 50-mL volumetric flask; 15 µL of the solution was quantitatively pipetted into a derivatization tube. The sample was dried under vacuum and oxidized with performic acid for the conversion of cysteine and cystine into cysteic acid; this was followed by vapor phase acidic hydrolysis using a boiling HCl solution at 110°C for 24 h. The sample was combined with alpha-amino butyric acid as an internal standard and then analyzed for cysteic acid using AccQ-Tag derivatization and HPLC (25, 26).

### Tryptophan analysis

A total of 10 mL of 4.2 M NaOH solution was added to a hydrolysis tube that contained 3 mL of human milk. In addition, 800 µL of 5-methyl-tryptophan was added to the hydrolysis tube as an internal standard. Following vacuum and nitrogen flush repeated three times, the tube was sealed under vacuum and placed in an oven at 110°C for 20 h to hydrolyze the sample. Following adjustment to pH 4.2 using 12 M HCl, centrifugation, and filtration, tryptophan was determined using reversed-phase HPLC.

### Nitrogen analysis

Total nitrogen was determined by complete combustion of each human milk sample using the LECO FP-528 nitrogen analyzer (LECO Corporation, St. Joseph, MI). An infant formula standard (NIST infant formula reference material 1846) solution with a nitrogen content of 0.221% (w/w) was used as the calibration standard.

### Protein and nitrogen calculations

Total protein content was calculated from total nitrogen as follows:

Total protein=total nitrogen×6.25.

True protein, including protein, free amino acids, and peptides, was calculated from total amino acids as follows:

True protein=total amino acids×100/116.

This calculation corrects the amino acid sum to a corresponding weight of polypeptide. Specifically, 100 g of protein (milk source) generates approximately 116 g of hydrolyzed amino acids due to water molecules added during protein hydrolysis (5, 27). Thus, true protein is a calculation of the amino acid sum, corrected for water added during hydrolysis to individual amino acids.

The percentage of protein nitrogen was calculated as true protein divided by total protein. Non-protein nitrogen was calculated as the amount remaining after subtracting the percentage of protein nitrogen from 100.

### Statistical analysis

Descriptive statistics were calculated for all variables, including individual amino acids, total amino acids, and total protein. An ANOVA model was used in the assessment of total protein and amino acid concentrations with the stage of lactation, country, mother's age and parity as covariates. *Post-hoc* pair-wise comparisons for total protein and total and individual amino acid concentrations in different countries were done by the Fisher's LSD test; as this was an exploratory analysis, no adjustments were made for multiple comparisons.

## Results

### Study population

**Fig. 1:**
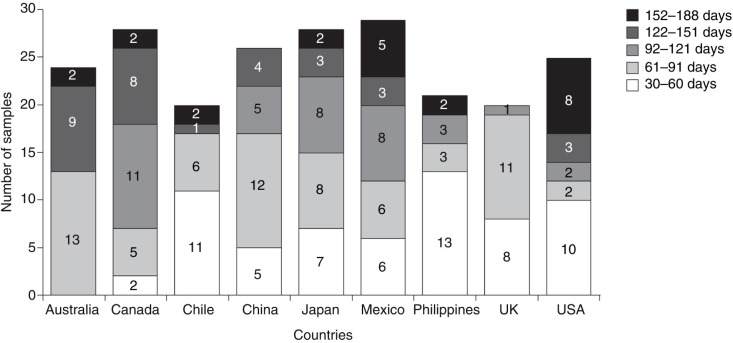
Distribution of human milk samples by country and stage of lactation.

Between 20 and 28 human milk samples were analyzed from each of the nine countries included in the study population (Fig. 1). The number of samples varied by the stage of lactation: *n*=62 for lactation days 30–60; *n*=66 for lactation days 61–91; *n*=38 for lactation days 92–121; *n*=31 for lactation days 122–151; and *n*=23 for lactation days 152–188. The mean (standard deviation [SD]) age of the mothers who provided the samples was 30 (4.8) years; median (range) parity was 1 (1–4) (Table 2).

**Table 2:** Demographics of study population per country

	Australia	Canada	Chile	China	Japan	Mexico	Philippines	UK	USA	All
Infants' age (Means)										
Days (range)	104.7 (68–169)	109.1 (60–163)	65.3 (30–183)	82.1 (33–149)	86.9 (30–188)	99.6 (34–160)	70.6 (30–183)	68.0 (42–114)	103.4 (35–188)	89.4 (30–188)
Mother's age (Means)										
Years (range)	30.2 (20–36)	32.8 (24–38)	25.7 (18–40)	27.5 (21–34)	31.1 (25–39)	31.1 (25–39)	27.3 (20–36)	32.4 (21–37)	30.6 (21–38)	30.0 (18–40)
Parity										
Median (range)	2 (1–3)	2 (1–4)	2 (1–4)	1 (1–2)	1 (1–3)	1 (1–3)	2 (1–4)	2 (1–4)	1 (1–3)	1 (1–4)
Race	100% Caucasian	86% Caucasian	100% Caucasian	100% Asian	100% Asian	89% Caucasian	100% Asian	100% Caucasian	84% Caucasian	–

### Protein and amino acid concentrations in the overall study population

**Fig. 2:**
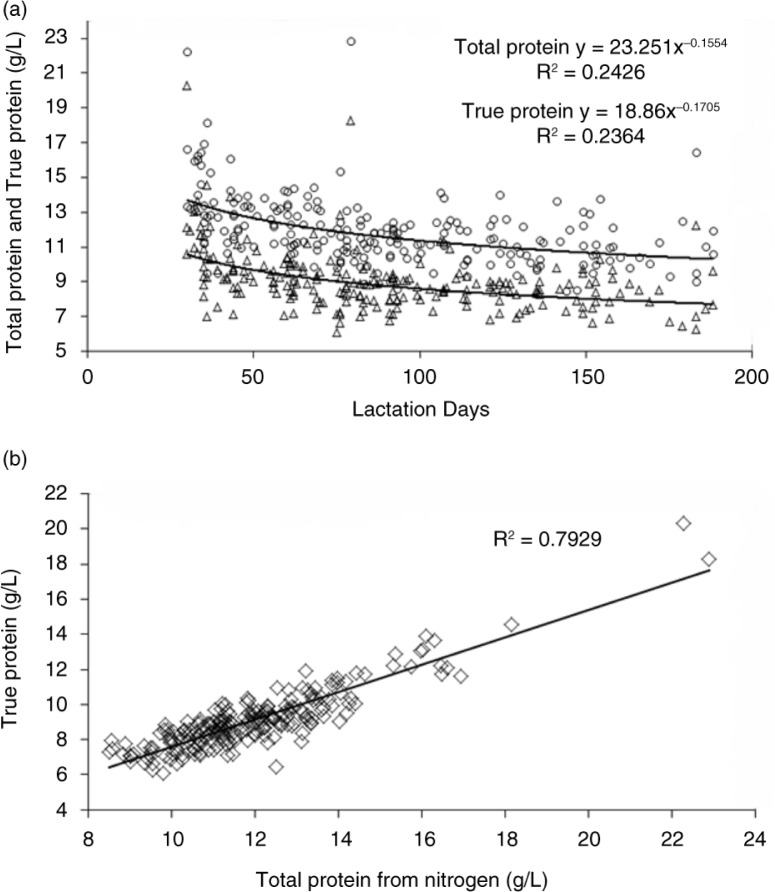
(a) Total protein and true protein over the course of lactation. (b) Correlation between total protein and true protein concentrations.

Table 3 summarizes the mean concentrations of amino acids, total protein, and true protein in human milk samples from the overall study population and by stage of lactation. The mean (SD) total protein concentration across all samples was 1,192 (200.9) mg/dL, and the true protein concentration across all samples was 908 (176.2) mg/dL. Overall, the mean concentration of true protein was 76% of the total protein concentration. Mean (SD) total amino acid concentration was 1,053 (204.4) mg/dL. As expected, the true protein concentration and total protein concentration were highly correlated (*R*^2^=0.7929) (Fig. 2). Results from multivariable analysis of variance (Table 4) demonstrated that the stage of lactation was significantly correlated with total protein concentration (*P*<0.0001) and total amino acid concentration (*P*<0.0001). Correlations with other variables (that is, the country, mother's age, and parity) were not statistically significant for either total protein or total amino acid concentration.

**Table 3:** Mean concentrations of amino acids, total protein, and true protein in human milk samples from overall study population (*n*=220) and in samples by stage of lactation

	All samples	Stage of lactation				
		
	30–188 days (*n*=220)	30–60 days (*n*=62)	61–91 days (*n*=66)	92–121 days (*n*=38)	122–151 days (*n*=31)	152–188 days (*n*=23)
Amino acid: essential, mg/dL						
CYS	23 (6.4)	27 (7.6)	22 (6.3)	21 (3.2)	19 (3.1)	21 (4.8)
HIS	26 (5.7)	30 (6.7)	25 (5.4)	25 (3.7)	24 (2.4)	23 (4.4)
ILE	59 (10.4)	67 (12.0)*	58 (9.3)	57 (7.0)	54 (5.1)	53 (8.4)
LEU	107 (20.6)	121 (24.5)*	104 (19.6)	104 (12.5)	99 (9.9)	97 (16.1)
LYS	72 (13.7)	81 (15.2)*	70 (13.2)	69 (9.4)	66 (7.0)	65 (11.0)
MET	17 (3.8)	19 (4.0)*	16 (3.6)	16 (3.3)	15 (2.1)	15 (2.9)
PHE	41 (10.4)	48 (12.7)	40 (10.5)	39 (5.1)	37 (4.1)	37 (7.6)
THR	49 (11.4)	56 (14.1)*	48 (11.5)	46 (5.4)	44 (4.4)	44 (8.9)
TRP	21 (4.8)	24 (5.7)*	21 (4.6)	19 (2.5)	18 (2.4)	18 (3.3)
TYR	48 (11.1)	54 (13.7)	47 (10.7)	45 (5.6)	43 (4.7)	42 (8.7)
VAL	60 (12.2)	69 (14.6)*	59 (11.6)	58 (7.0)	55 (5.8)	54 (9.5)
Amino acid: non-essential, mg/dL						
ALA	40 (9.4)	46 (11.4)	39 (9.4)	38 (4.9)	37 (4.3)	37 (7.3)
ARG	42 (13.2)	49 (16.4)	41 (14.5)	40 (5.3)	38 (4.9)	39 (10.2)
ASP	90 (18.9)	103 (22.5)*	88 (17.9)	85 (11.6)	83 (9.2)	81 (14.7)
GLU	187 (26.1)	201 (28.9)	184 (25.5)	186 (19.9)	179 (15.8)	173 (25.4)
GLY	25 (8.2)	29 (9.6)	24 (9.5)	24 (3.7)	23 (2.9)	22 (5.9)
PRO	95 (16.6)	107 (19.0)*	92 (15.3)	93 (10.7)	88 (8.8)	87 (13.3)
SER	50 (12.8)	57 (15.9)	48 (13.0)	47 (6.6)	45 (5.1)	44 (9.4)
Total amino acids, mg/dL	1,053 (204.4)	1,188 (241.6)*	1,026 (197.8)	1,013 (117.8)	968 (92.0)	953 (163.0)
Total protein, mg/dL	1,192 (200.9)	1,337 (211.4)*	1,166 (193.9)	1,132 (120.9)	1,103 (142.8)	1,097 (162.1)
True protein, mg/dL	908 (176.2)	1,024 (208.3)*	884 (170.5)	873 (101.6)	834 (79.3)	821 (140.5)
NPN, %	24 (6.6)	24 (0.8)	24 (0.8)	23 (0.9)	24 (1.2)	25 (1.6)
PN, %	76 (6.6)	76 (0.8)	76 (0.8)	77 (0.9)	76 (1.2)	75 (1.6)

NPN, non-protein nitrogen; PN, protein nitrogen; SD, standard deviation.

Data are mean (SD).

Total protein is calculated using total nitrogen×a protein factor of 6.25.

True protein is calculated from the total amino acid concentration.

* *P*<0.0001 30–60 days versus following 4 months.

**Table 4:** Analysis of variance results for total protein concentration and total amino acid concentration

	*P*	Variation (% contribution)
Total protein concentration		
Stage of lactation	<0.0001	22.9
Country	0.1498	5.2
Mother's age	0.3168	0.4
Parity	0.9348	0.2
Total amino acid concentration		
Stage of lactation	<0.0001	16.9
Country	0.0517	5.9
Mother's age	0.5726	0.1
Parity	0.9194	0.2

Variability in the individual amino acid concentrations, assessed by the coefficient of variation (CV), ranged from 14 to 32% for absolute concentrations (mg/dL) of amino acids in the 220 samples; the mean CV was 23% (Table 5). When normalized according to the percentage of total amino acids, the CVs were much lower, ranging from 3 to 13%, with a mean of 7%.

**Table 5:** Variation in the content of individual amino acids in 220 human milk samples from nine countries

Amino acid	Mean, mg/dL	Coefficient of variation (%)	Mean (% of amino acid)	Coefficient of variation (%)
Essential				
CYS	23	28	2.1	13
HIS	26	22	2.5	5
ILE	59	18	5.7	5
LEU	107	19	10.2	3
LYS	72	19	6.8	4
MET	17	23	1.6	11
PHE	41	25	3.9	5
THR	49	23	4.6	4
TRP	21	23	2	11
TYR	48	23	4.5	5
VAL	60	20	5.7	3
Non-essential				
ALA	40	23	3.8	6
ARG	42	31	4	12
ASP	90	21	8.5	4
GLU	187	14	17.9	7
GLY	25	32	2.4	11
PRO	95	17	9.1	6
SER	50	26	4.7	6
Total	1,053	19	100	0
Mean		23		7

### Protein and amino acid concentrations by stage of lactation

**Fig. 3:**
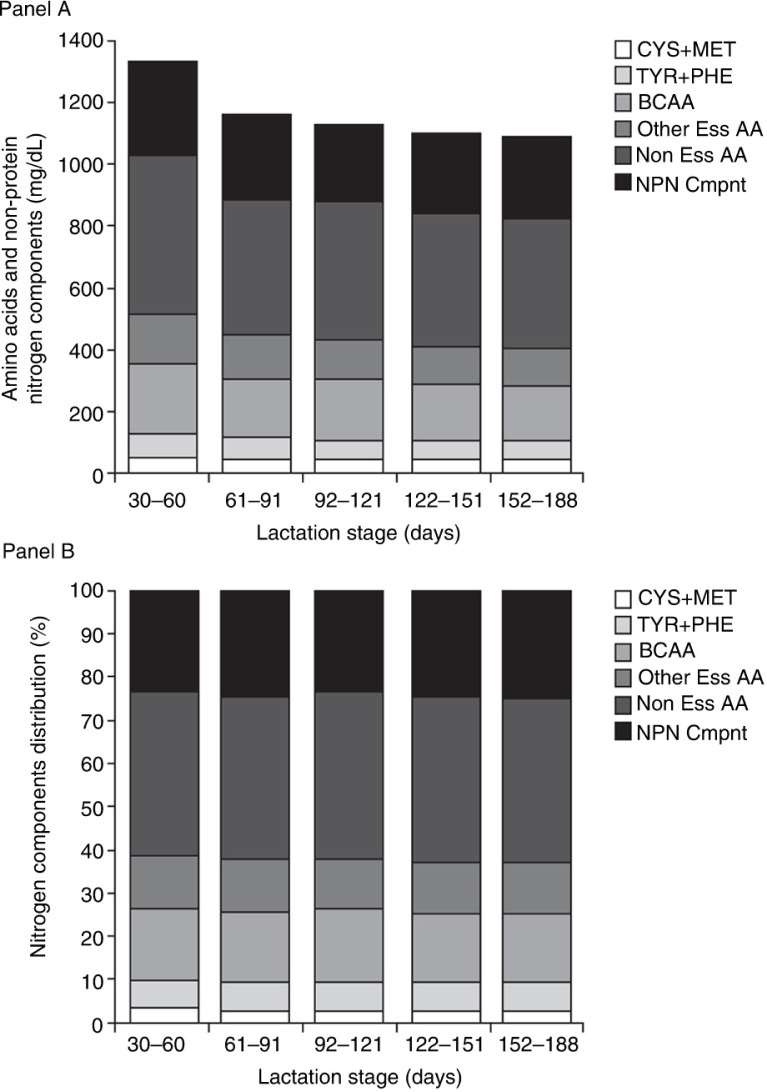
(a) Decline in total protein (or nitrogen-containing components) and each amino acid group and non-protein nitrogen components by stage of lactation. (b) Relative contributions of individual amino acid groups and non-protein-containing components to total protein content by stage of lactation. BCAA, branched-chain amino acids; Other Ess AA, other essential amino acids; Non Ess AA, non-essential amino acids; NPN Cmpnt, non-protein nitrogen components.

The mean concentrations of total protein, true protein, total amino acids, and most individual amino acids in human milk declined steadily from 30 to 188 days of lactation (Table 3). The total protein concentration and total amino acid concentration were both significantly higher (*P*<0.0001) in the second month of lactation (days 30–60) compared with the following 4 months. In addition, the total amino acid concentration was significantly higher (*P*=0.029) in the third month of lactation (days 61–91) as compared with the sixth month (days 152–188). The decline in total protein that occurred from the second month of lactation through the sixth month reflected nearly equal declines in the various components of total protein (Fig. 3a). As such, despite steady declines, the proportion of essential amino acids, non-essential amino acids, and non-protein nitrogen components remained relatively unchanged as the duration of lactation increased (Fig. 3b). Similarly, with the exception of cysteine and glutamic acid, the relative contributions of each individual amino acid to total amino acids remained consistent between lactation months 2 and 6 (Table 6).

**Table 6:** Amino acid profile by stage of lactation

	Amino acids as proportion of total amino acids, mean % (SD)					
		
Amino acid	30–60 days (*n*=67)	61–91 days (*n*=74)	92–121 days (*n*=40)	122–151 days (*n*=34)	152–188 days (*n*=25)	P*
Essential						
CYS	2.2 (0.30)	2.1 (0.28)	2.1 (0.22)	2.0 (0.25)	2.1 (0.22)	<0.01
HIS	2.5 (0.17)	2.5 (0.13)	2.5 (0.12)	2.5 (0.08)	2.4 (0.10)	NS
ILE	5.6 (0.34)	5.7 (0.30)	5.7 (0.17)	5.6 (0.22)	5.6 (0.22)	NS
LEU	10.2 (0.35)	10.2 (0.32)	10.2 (0.28)	10.2 (0.33)	10.2 (0.23)	NS
LYS	6.8 (0.30)	6.8 (0.31)	6.8 (0.31)	6.7 (0.28)	6.8 (0.28)	NS
MET	1.7 (0.16)	1.6 (0.18)	1.5 (0.24)	1.6 (0.11)	1.6 (0.15)	NS
PHE	4.0 (0.22)	3.9 (0.21)	3.9 (0.16)	3.9 (0.12)	3.9 (0.16)	NS
THR	4.7 (0.23)	4.7 (0.21)	4.6 (0.12)	4.6 (0.12)	4.6 (0.20)	NS
TRP	2.0 (0.24)	2.0 (0.22)	1.9 (0.17)	1.9 (0.15)	1.9 (0.18)	NS
TYR	4.6 (0.25)	4.6 (0.22)	4.5 (0.17)	4.5 (0.17)	4.4 (0.25)	NS
VAL	5.8 (0.17)	5.7 (0.16)	5.7 (0.14)	5.7 (0.19)	5.7 (0.14)	NS
Non-essential						
ALA	3.9 (0.26)	3.8 (0.25)	3.8 (0.13)	3.8 (0.15)	3.8 (0.17)	NS
ARG	4.1 (0.51)	4.0 (0.52)	3.9 (0.28)	4.0 (0.31)	4.1 (0.48)	NS
ASP	8.6 (0.45)	8.5 (0.38)	8.4 (0.31)	8.5 (0.32)	8.5 (0.32)	NS
GLU	17.0 (1.22)	17.9 (1.17)	18.3 (0.92)	18.4 (0.92)	18.1 (1.08)	<0.01
GLY	2.5 (0.29)	2.4 (0.32)	2.4 (0.20)	2.4 (0.14)	2.3 (0.25)	NS
PRO	9.1 (0.65)	9.1 (0.66)	9.2 (0.40)	9.1 (0.46)	9.2 (0.46)	NS
SER	4.8 (0.40)	4.7 (0.29)	4.7 (0.23)	4.7 (0.16)	4.6 (0.25)	NS

NS, not statistically significant; SD, standard deviation.

* *P*<0.01, 30–60 days versus following 4 months.

### Protein and amino acid concentrations by country

**Fig. 4:**
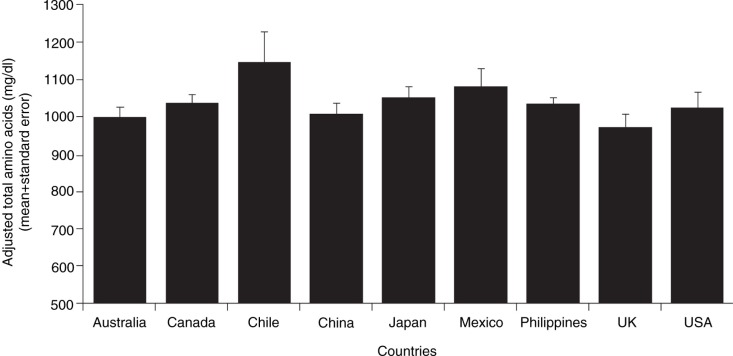
Adjusted total amino acid concentration by country. Data were adjusted for the stage of lactation as the number of samples was unevenly distributed across lactation stages due to limited sample availability.The mean total amino acid concentration in human milk samples from Chile was significantly higher than the mean concentration in samples from Australia, China, the Philippines, the United Kingdom, and the United States (*P*<0.02).

Mean concentrations of amino acids, total protein, and true protein in human milk samples by country were summarized (Table 7). The mean (SD) total protein concentration in human milk per individual countries ranged from 1,133 (125.5) to 1,366 (341.4) mg/dL. Protein and amino acid concentrations were similar across countries, and the overall effect of the country on the levels of total protein and total amino acids was not statistically significant, with the exception of Chile. Statistical analyses were performed after adjusting for the mother's age and stage of lactation, as the numbers of samples were unevenly distributed across lactation stages. *Post-hoc* comparisons between Chile and all the other countries were performed to evaluate the significance of the higher protein and amino acid levels in Chile. Even with adjustment, the results of these analyses showed that total protein, total amino acids, and most individual amino acid concentrations were significantly higher in the human milk samples from Chile as compared with the mean concentrations in samples from Australia, China, the Philippines, the United Kingdom, and the United States (*P*<0.05 for each comparison) (Fig. 4).

**Table 7:** Mean concentrations of amino acids, total protein, and true protein in human milk samples by country

	Australia (*n*=24)	Canada (*n*=28)	Chile (*n*=20)	China (*n*=26)	Japan (*n*=28)	Mexico (*n*=28)	Philippines (*n*=21)	UK (*n*=20)	USA (*n*=25)	All (*n*=220)
Amino acids: essential, mg/dL										
CYS	21 (4.3)	21 (3.2)	30 (11.6)	21 (5.0)	21 (4.0)	23 (8.8)	23 (4.7)	23 (3.2)	22 (4.7)	23 (6.4)
HIS	24 (3.4)	25 (2.5)	31 (10.0)	26 (4.4)	27 (4.6)	27 (7.0)	27 (4.5)	25 (4.1)	24 (6.0)	26 (5.7)
ILE	56 (7.5)	57 (5.3)	66 (16.6)	59 (10.0)	61 (10.8)	59 (10.2)	60 (9.0)	59 (9.0)	59 (12.2)	59 (10.4)
LEU	100 (13.9)	104 (10.0)	121 (37.8)	107 (17.3)	110 (19.2)	109 (23.5)	108 (15.0)	104 (16.3)	107 (21.2)	107 (20.6)
LYS	67 (10.8)	69 (7.4)	82 (22.9)	69 (10.9)	74 (11.8)	72 (15.8)	71 (9.9)	72 (11.4)	72 (15.3)	72 (13.7)
MET	16 (2.4)	16 (2.1)	19 (6.6)	16 (4.0)	17 (3.6)	17 (4.6)	17 (3.6)	16 (3.0)	17 (3.3)	17 (3.8)
PHE	38 (6.3)	40 (4.5)	51 (20.8)	40 (7.1)	42 (7.8)	42 (14.5)	41 (5.9)	40 (6.3)	40 (8.3)	41 (10.4)
THR	45 (6.6)	47 (4.6)	60 (23.0)	47 (7.7)	49 (7.7)	50 (15.7)	48 (7.0)	48 (7.2)	47 (9.5)	49 (11.4)
TRP	19 (3.2)	20 (2.2)	26 (8.8)	19 (3.4)	20 (3.3)	21 (6.5)	20 (3.0)	23 (3.3)	21 (3.4)	21 (4.8)
TYR	44 (6.5)	46 (4.8)	57 (22.1)	47 (8.2)	48 (9.0)	48 (14.1)	47 (7.1)	46 (7.7)	47 (9.7)	48 (11.1)
VAL	55 (7.7)	58 (5.8)	71 (23.5)	60 (9.1)	62 (10.0)	61 (15.0)	61 (8.7)	59 (8.6)	59 (11.0)	60 (12.2)
Amino acids: non-essential, mg/dL										
ALA	37 (6.2)	39 (4.6)	50 (17.9)	39 (6.3)	40 (6.5)	42 (12.8)	39 (5.4)	40 (6.2)	40 (7.4)	40 (9.4)
ARG	39 (7.8)	40 (5.4)	56 (26.3)	41 (8.1)	42 (7.5)	44 (20.2)	40 (5.2)	39 (7.6)	41 (10)	42 (13.2)
ASP	83 (13.9)	87 (9.7)	107 (33.9)	87 (14.2)	92 (14.4)	92 (23.4)	86 (13.6)	89 (13.8)	90 (18.8)	90 (18.9)
GLU	178 (23.7)	186 (15.1)	198 (40.5)	183 (23.3)	192 (25.1)	191 (25.1)	180 (22.6)	186 (22.3)	191 (31.6)	187 (26.1)
GLY	23 (4.4)	24 (3.3)	33 (15.9)	24 (4.0)	25 (4.8)	27 (13.7)	25 (4.1)	25 (4.2)	23 (5.2)	25 (8.2)
PRO	88 (11.2)	92 (9.3)	104 (26.8)	96 (16.2)	98 (16.9)	95 (15.5)	100 (15.2)	92 (15.0)	95 (18.1)	95 (16.6)
SER	46 (7.4)	47 (5.2)	62 (25.4)	48 (7.8)	51 (9.4)	51 (17.6)	50 (9.2)	48 (7.7)	47 (10.8)	50 (12.8)
Total, mg/dL	978 (139.0)	1,016 (94.3)	1,224 (382.5)	1,029 (159.1)	1,070 (165.9)	1,071 (252.1)	1,045 (141.9)	1,034 (148.8)	1,043 (200.3)	1,053 (204.4)
Total protein, mg/dL	1,145 (164.0)	1,133 (125.5)	1,366 (341.4)	1,167 (149.7)	1,205 (160.1)	1,179 (269.2)	1,191 (143.7)	1,215 (139.6)	1,173 (180.9)	1,192 (200.9)
True protein, mg/dL	843 (119.8)	876 (81.3)	1,055 (329.7)	887 (137.2)	922 (143.1)	923 (217.3)	901 (122.4)	892 (128.2)	899 (172.7)	908 (176.2)
NPN,%	26 (7.3)	22 (5.8)	24 (6.8)	24 (5.9)	23 (6.2)	22 (5.0)	24 (5.7)	27 (6.9)	23 (8.8)	24 (6.6)
PN,%	74 (7.3)	78 (5.8)	76 (6.8)	76 (5.9)	77 (6.2)	78 (5.0)	76 (5.7)	73 (6.9)	77 (8.8)	76 (6.6)

NPN, non-protein nitrogen; PN, protein nitrogen; SD, standard deviation.

Data reflect mean (SD).

Total protein is calculated using total nitrogen×a protein factor of 6.25.

True protein is calculated from the total amino acid concentration.

## Discussion

Human milk has a substantially lower total protein concentration than the milks of other species. However, human milk provides a richer source of essential amino acids, which allows infants to meet their protein requirements in a lower concentration (28). The unique quantity and quality of proteins in human milk are important because of the elevated requirements for essential amino acids and the conditionally essential nature of certain other amino acids during infancy (29).

Human milk changes in both protein content and whey-to-casein composition over the course of lactation. During the first 30 days of lactation, the decline in protein content and the compositional shift in whey-to-casein ratio is clearly apparent. Early milk has a whey-to-casein ratio of approximately 90:10 in early milk, which evolves to approximately 50:50 in late lactation (30, 31); however, beyond the first month, the rate of change in protein content and composition becomes less obvious. It has been estimated that, during infancy, when protein accretion is at its highest, essential amino acids make up one-fifth of protein requirements. By comparison, later in childhood, essential amino acids comprise one-fifth of protein requirements and reflect only one-tenth of the protein requirements in adults (32). Thus, the amino acid composition of human milk has relevance for understanding the nutritional needs of infants. A further measure of nutritional value is true protein, which represents only the polypeptide portion of total protein. (The ‘Methods’ section includes the calculation of true protein).

Protein and amino acid analyses in this study included samples from the second to the sixth month of lactation because of the known changes in human milk composition during the first month postpartum. The data from this study show a relatively large variation of amino acids and protein concentration among mothers’ milk samples. However, the variations in amino acid content, protein content, protein nitrogen, and non-protein nitrogen composition observed in this study are consistent with other human milk studies (17, 19). When the absolute concentrations of the individual amino acids were normalized to percentage of total amino acids, the variation in the data decreased considerably, indicating high consistency in the amino acid profile of human milk with very little impact from mother's race/ethnicity or age. Larger CVs in the amino acid profile (the percentage of amino acids) for cysteine and tryptophan may be explained by the use of separate procedures for the analysis of these two amino acids. The higher CVs in both the amino acid amount and the profile for methionine, arginine, and glycine may be due to the susceptibility of methionine and glycine to oxidation under hydrolysis conditions; the oxidized product of methionine may have affected the integration and quantification of arginine in the analysis method.

**Fig. 5:**
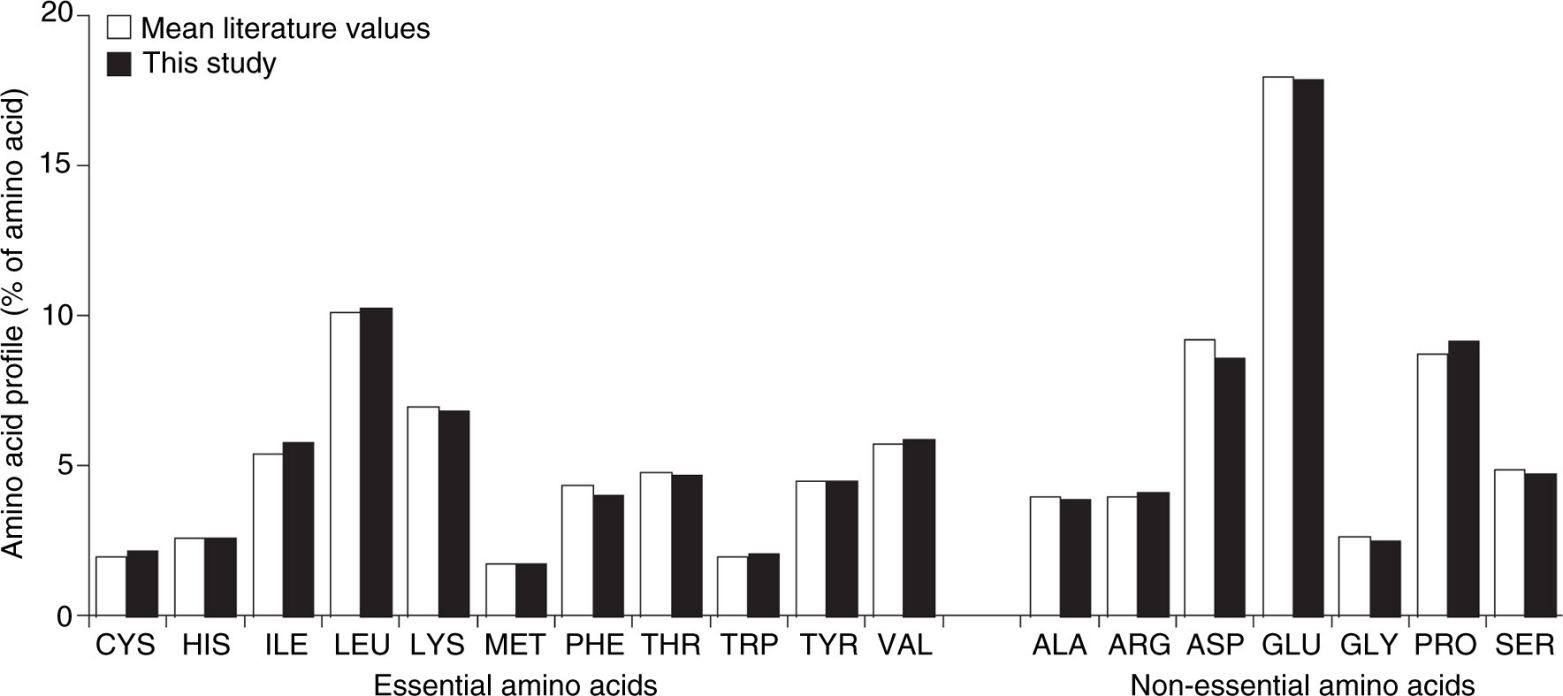
A comparison of the amino acid profile from this nine-country study with mean values reported in the literature, as detailed in Table 1 (5–21).

Over the years, the Institute of Medicine of the US National Academy of Sciences has organized scientific expert panels to evaluate the totality of scientific literature on individual nutrients and publish Dietary Recommended Intakes for macronutrients and micronutrients for all age groups. The recommendation for the protein intake for infants during the first 6 months of life is based on the average volume of human milk (0.78 L/day) consumed by infants during this age range at the average protein content of human milk (11.7 g/L), as determined from data from several studies conducted in the United States using various methods of analysis (2). The mean of protein content of human milk from US-based studies is consistent with results described in this paper, which represent a more global analysis. Moreover, the amino acid profile reported in the current study is similar to the calculated mean based on references in the literature, which included amino acid composition of human milk (Fig. 5) (5–19). The mean total protein and amino acid concentrations reported here are also consistent with previously reported data (6, 12, 14, 15, 17, 18, 20).

The human milk samples from Chile contained significantly higher amounts of each amino acid, total amino acids, and protein than most of the other eight countries. These findings are consistent with the results of an analysis of lactoferrin levels in human milk samples from the same study, which found that the mean lactoferrin concentration was significantly higher in samples from Chile compared with the samples from the other eight countries (33), as were levels of zinc (34). Additionally, and also from this same study, the alpha-lactalbumin (a protein fraction) concentration throughout lactation differed between Chile and the other countries (35). The total amino acid concentration and total protein concentration were, however, highly correlated in the samples from Chile just as in the other countries. Thus, we cannot speculate why total protein levels would differ in milks from mothers in Chile versus other countries.

### Assessment of maternal diet

The maternal dietary intake of many important micronutrients has been shown to influence the concentration of those nutrients in human milk including vitamins A and E (36), fatty acids such as DHA (24), and carotenoids such as lutein and beta-carotene (23); however, the protein content of human milk has been shown to be relatively unaffected by maternal diet (36, 37). This lack of influence was one of the reasons we were interested in evaluating protein content across countries and explains why we did not evaluate maternal dietary protein intake in this study.

This study provides considerable evidence that the protein content and amino acid composition of human milk are relatively uniform across geographic regions when compared by stage of lactation. Our amino acid analysis revealed little change in amino acid profiles over time. Only cysteine and glutamic acid showed any significant variation with stage of lactation. The shift in these amino acids would be consistent with an increase in the proportion of casein as lactation continues because the concentration of cysteine is lower (and glutamic acid is higher) in the casein fraction of human milk, compared with the whey fraction.

In summary, the results from this large-scale, multinational study of 220 human milk samples revealed a high level of uniformity in protein and amino acid composition across a wide range of geographic locations. With the exception of Chile, there were no significant differences between countries in amino acid, true protein, and total protein concentrations. In all nine countries included in the study, protein and amino acid concentrations declined steadily from 30 to 188 days postpartum. Moreover, the proportion of true protein and the amino acid profiles of human milk were generally consistent across lactation stages and countries. Several features of this study, including the size and diversity of the study population, and our use of highly standardized procedures for collection, storage, and analysis of human milk samples strengthen the validity of our findings and enhance their applicability.

## References

[1] World Health Organization (2003). Global strategy for infant and young children feeding.

[2] Food and Nutrition Board, Institute of Medicine, National Academy of Sciences (2005). Dietary reference intakes for energy, carbohydrate, fiber, fat, fatty acids, cholesterol, protein, and amino acids (macronutrients).

[3] Cheung MW, Pratt EL, Fowler DI (1953). Total amino acid composition in mature human milk analysis by the ion exchange resin column chromatographic technic. Pediatrics.

[4] Spackman DH, Stein WH, Moore S (1958). Automatic recording apparatus for use in the chromatography of amino acids. Anal Chem.

[5] Lonnerdal B, Forsum E, Hambraeus L (1976). The protein content of human milk. Nutr Rep Int.

[6] Raiha N, Minoli I, Moro G, Bremer HJ (1986). Milk protein intake in the term infant. II. Effects on plasma amino acid concentrations. Acta Pediatr Scand.

[7] Picone TA, Benson JD, Moro G, Minoli I, Fulconis F, Rassin DK (1989). Growth, serum biochemistries, and amino acids of term infants fed formulas with amino acid and protein concentrations similar to human milk. J Pediatr Gastroenterol Nutr.

[8] Atkinson SA, Anderson GH, Bryan MH (1980). Human milk: comparison of the nitrogen composition from mothers of premature and full-term infants. Am J Clin Nutr.

[9] Renner E (1983). Milk and dairy products in human nutrition.

[10] United States Department of Agriculture, Agricultural Research Service, Nutrient Data Laboratory USDA National Nutrient Database for Standard Reference, Release 28.

[11] Svanberg U, Gebre-Medhin M, Ljungqvist B, Olsson M (1977). Breast milk composition in Ethiopian and Swedish mothers. III. Amino acids and other nitrogenous substances. Am J Clin Nutr.

[12] Jarvenpaa AL, Rassin DK, Raiha NCR, Gaull GE (1982). Milk proteins quantity and quality in the term infant. II. Effects on acidic and neutral amino acids. Pediatrics.

[13] Harzer G, Bindels JG, M Xanthou (1987). Main compositional criteria of human milk and their implications on nutrition in early infancy. New aspects of nutrition in pregnancy, infancy and prematurity.

[14] Donovan SM, Lonnerdal B (1989). Non-protein nitrogen and true protein in infant formulas. Acta Pediatr Scand.

[15] Hanning RM, Paes B, Atkinson SA (1992). Protein metabolism and growth of term infants in response to a reduced-protein, 40:60 whey: casein formula with added tryptophan. Am J Clin Nutr.

[16] Davis TA, Nguyen HV, Garcia-Bravo R, Fiorotto ML, Jackson EM, Lewis DS (1994). Amino acid composition of human milk is not unique. J Nutr.

[17] Motil KJ, Thotathuchery M, Bahar A, Montandon CM (1995). Marginal dietary protein restriction reduced nonprotein nitrogen, but not protein nitrogen, components of human milk. J Am Coll Nutr.

[18] Darragh AJ (1998). The amino acid composition of human milk corrected for amino acid digestibility. Br J Nutr.

[19] Zhao X, Xu Z, Wang Y, Sun Y (1989). [Studies of the relation between the nutritional status of lactating mothers and milk composition as well as the milk intake and growth of their infants in Beijing. Pt. 4. The protein and amino acid content of breast milk]. Ying Yang Xue Bao.

[20] Wu ZC, Chijang CC, Lau BH, Hwang B, Sugawara M, Idota T (2000). Crude protein content and amino acid composition in Taiwanese human milk. J Nutr Sci Vitaminol.

[21] Lonnerdal B, Forsum E, Hambraeus L (1976). A longitudinal study of protein, nitrogen and lactose contents of human milk from Swedish well-nourished mothers. Am J Clin Nutr.

[22] Prentice A, RG Jensen (1995). Regional variations in the composition of human milk. Handbook of milk composition.

[23] Canfield LM, Clandinin MT, Davies DP, Fernandez MC, Jackson J, Hawkes J (2003). Multinational study of major breast milk carotenoids of healthy mothers. Eur J Nutr.

[24] Yuhas B, Pramuk K, Lien EL (2006). Human milk fatty acid composition from nine countries varies most in DHA. Lipids.

[25] Waters Corporation (2008). AccQ-fluor reagent kit care and use manual.

[26] Liu HJ, Chang BY, Yan HW, Yu FH, Liu XX (1995). Determination of amino acids in food and feed by derivatization with 6-aminoquinolyl-N-hydroxysuccinimidyl carbamate and reversed-phase liquid chromatographic separation. J AOAC Int.

[27] Soupart P, Moore S, Bigwood EJ (1954). Amino acid composition of human milk. J Biol Chem.

[28] Heine WE, Klein PD, Reeds PJ (1991). The importance of alpha-lactalbumin in infant nutrition. J Nutr.

[29] Heird WC, BA Bowman, RM Russell (2006). Infant nutrition. Present knowledge in nutrition.

[30] Kunz C, Lonnerdal B (1992). Re-evaluation of the whey protein/casein ratio of human milk. Acta Paediatr.

[31] Lonnerdal B (2003). Nutritional and physiologic significance of human milk proteins. Am J Clin Nutr.

[32] Young VR (1994). Adult amino acid requirements: the case for major revision in current recommendations. J Nutr.

[33] Lien E, Jackson J, Kuhlman C, Pramuk K, Lönnerdal B, Janszen D (2004). Variations in concentrations of lactoferrin in human milk: a nine country survey. Adv Exp Med Biol.

[34] Radzanowski GM, Jackson JK, Pramuk K, Kaup SM (1999). Comparison of trace elements and macronutrients in breast milk of women from eight different geographical locations.

[35] Jackson JG, Janszen DB, Lonnerdal B, Lien EL, Pramuk KP, Kuhlman CF (2004). A multinational study of alpha-lactalbumin concentrations in human milk. J Nutr Biochem.

[36] Lonnerdal B (1986). Effect of maternal dietary intake on human milk composition. J Nutr.

[37] Nommsen LA, Lovelady CA, Heinig MJ, Lonnerdal B, Dewey KG (1991). Determinants of energy, protein, lipid, and lactose concentrations in human milk during the first 12 months of lactation: the DARLING Study. Am J Clin Nutr.

